# Southern African HIV Clinicians’ Society gender-affirming healthcare guideline for South Africa

**DOI:** 10.4102/sajhivmed.v22i1.1299

**Published:** 2021-09-28

**Authors:** Anastacia Tomson, Chris/tine McLachlan, Camilla Wattrus, Kevin Adams, Ronald Addinall, Rutendo Bothma, Lauren Jankelowitz, Elliott Kotze, Zamasomi Luvuno, Nkanyiso Madlala, Savuka Matyila, Anil Padavatan, Mershen Pillay, Mmamontsheng D. Rakumakoe, Mathilde Tomson-Myburgh, Willem D.F. Venter, Elma de Vries

**Affiliations:** 1 My Family GP, Cape Town, South Africa; 2 Shemah Koleinu, Cape Town, South Africa; 3 KwaZulu-Natal Department of Health, Pietermaritzburg, South Africa; 4 Department of Psychology, College of Human Sciences, University of South Africa, Pretoria, South Africa; 5 Professional Association for Transgender Health South Africa, Cape Town, South Africa; 6 Psychological Society of South Africa, Johannesburg, South Africa; 7 Southern African HIV Clinicians Society, Johannesburg, South Africa; 8 Department of Plastic Surgery, Faculty of Health Science, University of Cape Town, Cape Town, South Africa; 9 Department of Social Development, Faculty of Humanities, University of Cape Town, Cape Town, South Africa; 10 Southern African Sexual Health Association, Cape Town, South Africa; 11 Wits Reproductive Health Institute, Johannesburg, South Africa; 12 Psychologist, Independent Practice, Cape Town, South Africa; 13 School of Nursing and Public Health, Centre for Rural Health, University of KwaZulu-Natal, Durban, South Africa; 14 Gender Dynamix, Cape Town, South Africa; 15 Department of Speech-Language Therapy, Faculty of Health Sciences, University of KwaZulu-Natal, Durban, South Africa; 16 Department of Speech-Language Therapy, Faculty of Health Sciences, Massey University, Auckland, New Zealand; 17 Quadcare, Johannesburg, South Africa; 18 Ezintsha, Faculty of Health Sciences, University of the Witwatersrand, Johannesburg, South Africa; 19 Cape Town Metro Health Services, Cape Town, South Africa; 20 School of Public Health and Family Medicine, Faculty of Health Science, University of Cape Town, Cape Town, South Africa

## Contents

Executive summary
Scope and purpose
Audience
Methods

1. Introduction
2. Informed consent
  2.1. The process
  2.2. Hormone therapy and surgery
  2.3. Children and adolescents
  2.4. Intellectual and developmental disability
3. Primary care
  3.1. The importance of the primary care provider
  3.2. Comprehensive care
    3.2.1. Violence
    3.2.2. Mental health
    3.2.3. Substance use
    3.2.4. Fertility and contraception
    3.2.5. Cancer
    3.2.6. Sexual health
    3.2.7. Sexually transmitted infections
    3.2.8. HIV
    3.2.9. Physical examination
4. Non-medical gender-affirming practices
  4.1. Binding
  4.2. Tucking
  4.3. Padding and packing
5. Psychosocial care
  5.1. The role of the mental healthcare provider
  5.2. Children
  5.3. Adolescents
  5.4. Adults
  5.5. The meso and macro context
6. Hormone therapy
  6.1. Background
  6.2. Indications
  6.3. Feminising therapy
  6.4. Masculinising therapy
  6.5. Adolescents
  6.6. Mature clients
7. Surgery
  7.1. Preoperative considerations
  7.2. Peri-surgical care
8. Institutions
  8.1. Care facilities
  8.2. Correctional facilities
  8.3. Work facilities
  8.4. Educational facilities
9. Voice and communication
10. Key terms

Acknowledgements
References
Appendix 1: The role of health professionals in change of gender marker at Home Affairs: Act 49
Appendix 2: Client information and consent form for feminising hormone therapy
Appendix 3: Client information and consent form for masculinising hormone therapy

## Executive summary

We support an affirming approach to managing the transgender and gender diverse (TGD) client, centering on the individual’s agency, autonomy and right to self-determination, as opposed to practices that pathologise and stigmatise transgender identity, imposing barriers to accessing healthcare services.

Transgender and gender diverse individuals have long faced discrimination on multiple axes, both globally and in South Africa. Although South Africa enshrines the protection of human rights in its Constitution, TGD individuals continue to face marginalisation, prejudice and threats to their safety. Challenges, including homelessness, unemployment, poor social support, bullying, harassment and violence, persist, indicating failures of policy development, practice implementation and a disregard for the human rights of individuals in the TGD community.

This guideline has been developed primarily with the intention of centering and amplifying voices of TGD individuals in order to facilitate access to healthcare that is sensitive, skilled and respectful. We recognise that there are significant gaps in the knowledge and skills of healthcare providers, and there is a lack of understanding of the unique experiences faced by TGD persons. The prevailing sentiment that many healthcare providers hold around TGD individuals, informed by ignorance and conditioning within social and societal structures, are malevolent towards this community, and often include harmful assumptions and generalisations. We believe that healthcare providers have an ethical obligation to interrogate these notions, and we promote an attitude of respect for diversity that upholds human rights.

It has been well established that access to competent and dignified gender-affirming healthcare (GAHC) is not only safe but also plays a significant role in improving measurable outcomes for TGD clients. It has also been well established that pathologising approaches and practices that limit access to care can be damaging and harmful.

Finally, we recognise that TGD individuals have historically endured being undermined, condescended to and pitied by the healthcare system and its providers. We affirm a commitment to upholding a strength-based perspective that values and respects the experiences of TGD clients and celebrates their individual identity rather than merely accepting or tolerating it.

This guideline, which no doubt will require ongoing revision, reflection and refinement in consultation with TGD communities and healthcare providers, represents a first step made in good faith towards creating a practical tool founded in robust scientific evidence, lodged within a human rights framework, and is intended to facilitate access to skilled and sensitive care that will yield tangible benefits to this unique and important group.

## Scope and purpose

- Provide evidence-informed best practice recommendations in order to enable South African healthcare providers, including psychosocial and allied healthcare professionals, to offer quality, affirming services to TGD clients. The term ‘client’, for the purposes of this guideline, includes service users, patients and participants.
- Provide support to TGD clients when accessing healthcare services.
- Note: this publication is a summary version of an expanded guideline, which can be accessed here: https://sahivsoc.org/Subheader/Index/sahcs-guidelines.

## Audience

This includes all healthcare providers, particularly those working in a primary care setting, public or private, or that care for TGD clients.

## Methods

The guideline development committee comprised 17 people, chaired by Dr Anastacia Tomson and Rev. Chris/tine McLachlan, which was inclusive, with representation of providers, advocates and civil society organisations in the TGD space, and many with personal experience as a TGD client. Development was predicated on the necessity to amplify the voices of those within the TGD community in order to better meet their needs, rather than presuming that healthcare providers can address those needs alone. This guideline was informed by evidence-based research studies, as well as provider experience from within the field. The committee worked from a gender-affirming, non-gatekeeping, depathologising perspective using a participatory approach that centres on the TGD client’s agency and humanity, and upholds their dignity.^1,2,3^ Strict values underpin this guideline, as shown in Table 1. In order to ensure applicability to the South African context, focused effort was made to review local research studies. Resources from the global South were then accessed, and only key resources from the global North were incorporated. An extensive, external peer review process was conducted, which included both health provider and community reviews. Guideline development and publication were supported by the Southern African HIV Clinicians’ Society (SAHCS) through Dr Camilla Wattrus and Dr Lauren Jankelowitz.

**TABLE 1:** Values underpinning this guideline.

Value	Description
Affirmation	We affirm and respect transgender and gender diverse (TGD) individuals, and acknowledge that the full spectrum of gender identities and diversities is valid.^3^ We understand that the concept of gender varies in relation to political and sociocultural contexts, personal and intergenerational trauma, and migrant and disability status.^4^ We avoid assuming links between the gender role, sexual orientation and gender identity based on traditional, binarised, cisnormative and heteronormative understandings of femininity and masculinity.^5,6^
Dignity	All people have the right to dignity,^7^ and this should be respected and protected. We acknowledge that people whose gender identity and gender expression fall outside of narrow societal norms are subject to stigma, discrimination and even violence. Competent and affirming healthcare upholds personal dignity and is fundamental to protecting and promoting the rights of all human beings.
Equity	We uphold the Yogyakarta principles that state all people are equal, should have equal access to their basic human rights, and no one should be subjected to unfair discrimination.^8^ The Yogyakarta principles +10 further stipulate the ‘right to freedom from criminalisation and sanction on the basis of sexual orientation, gender identity, gender expression or sex characteristics’.^9^ It is important to recognise that some people are subject to intersecting layers of discrimination, such as of race, socio-economic status, geographical area, age, health disparities, transphobia and homophobia, sexism and gender discrimination, and the oppression of women.^2^ It is the role of healthcare providers to challenge all forms of stigma, abuse, inequality and oppression directed at the TGD minority.^3^
Inclusion	TGD individuals should be included as equal partners in making decisions about their own bodies and healthcare, and also in broader decision-making regarding laws, policies and guidelines that have impact on their access to healthcare services.
Informed consent	Healthcare providers and clients are partners in making choices about medical treatment: the provider should inform the client of the risks and benefits, and the client should make an informed decision about their own healthcare based on the information provided.^10^ This approach respects a client’s agency over their own body, and is both clinically safe and ethically sound.
Ethical principles in healthcare	These include autonomy (self-determination and agency), non-maleficence (no harm), beneficence (benefitting the client with access) and justice (fairness and equity).^11^ The healthcare provider is ethically obligated to treat the TGD client with dignity and respect, and to facilitate access to care (both general and gender-affirming) without gatekeeping or judgement.
Ubuntu – the participatory approach	*Umuntu ngumuntu ngabantu* – A person is a person through other people. The African concept of Ubuntu is central to South Africa’s democracy. It calls on us to view the inherent humanity in all people and helps us to understand human interdependence within the indigenous context. All people need to be seen, recognised and affirmed as who they are in order to live fulfilling human lives.^12,13^
Batho Pele	These eight principles aim at enhancing the quality and accessibility of government services by improving efficiency and accountability They include consultation, service standards, access, courtesy, information, openness and transparency, redress and value for money.^13,14,15^
Trans giftedness	TGD individuals are generally resilient, self-aware, possess inner strength, and have unique perspectives and insights.^16^ This enables them to engage with the world of gender in new and diverse ways.^17^ We acknowledge this and utilise a strength-based perspective rather than condescending to, undermining or pitying TGD individuals.

TGD, transgender and gender diverse.

## 1. Introduction

I don’t think we really know what freedom is in South Africa. What will it take for me to have the freedom and safety to stand up in public and say, ‘I am gender fluid’? I don’t just feel marginalised, I feel like there is no space for me at all. (Personal communication with client, Durban, South Africa. 2020)

South Africa is a country with a progressive Constitution and Bill of Rights that provide for dignity, equality and access to healthcare services.^7^ This is echoed by the *South African Health Professions Act* and general ethical rules for Health Professionals^18^, Social Service Professions,^19^ the Constitutions of the Professional Association for Transgender Health in South Africa^20^ and Psychological Society of South Africa^21^, and the Department of Health’s Batho Pele principles.^22^ Despite this, many transgender and gender diverse (TGD) individuals struggle to access gender-affirming healthcare (GAHC) services in South Africa.

Gender-affirming healthcare attends holistically to a TGD individual’s mental, physical and social well-being, and health needs, whilst respecting their self-identified gender.^23^ Each individual has unique needs, and the gender-affirming process is rarely linear. This process may include social and medical elements, or none at all. A client with a non-binary identity may have unique and specific treatment goals:

Because for me, my transitioning is more spiritual than physical. I live as a woman every day. My nieces and my nephews, they call me mom, and finding peace within myself and being able to fight for them to have a representation of what love looks like makes me feel fulfilled. Your womanhood is within you more than what is here on the physicality.^24^

It is important to note that as a healthcare provider, withholding or delaying treatment is not a neutral action. It can have an impact on the client’s mental health conditions and, in adolescents, may have implications on what medical or surgical treatment is required later in life. Gatekeeping (delaying treatment until the healthcare provider feels a particular subjective degree of certainty) is potentially harmful.^11^

## 2. Informed consent

### 2.1. The process

Informed consent (IC) in GAHC is complex and nuanced.^25,26^ The IC process should empower the individual by upholding their autonomy and maintaining their integrity.^11^ Even in a supportive and affirming environment, there is often an unequal power relationship between the client and the healthcare provider.^27^ This can be distressing to the client and have a negative impact on their care.^28^ The client and healthcare provider should be collaborative partners in decision- making.^1,29^ The healthcare provider should inform the client of the risks and benefits of the various treatment options, thus enabling the client to make an informed decision about their own healthcare.^30^

### 2.2. Hormone therapy and surgery

A healthcare provider wanting to prescribe hormone therapy (HT) does not require a letter from a mental health provider (MHP) in order to do so, and they may perform the psychosocial assessment themselves if comfortable.^6,26^ For gender-affirming surgery, a documented process of thorough IC is essential and, ideally, should be performed together with a multidisciplinary team that includes a MHP. If the client is able to consent, their autonomy should be respected and facilitated,^6^ and it is recommended that in the case of an MHP writing a referral letter to a surgeon, this be written in collaboration with the client.^2^

We note that the World Professional Association for Transgender Health (WPATH) Standards of Care 7^6^ states that a client should have two independent psychological evaluations prior to surgery. However, it has been convincingly argued that this is not necessary for all clients.^31^

### 2.3. Children and adolescents

The Children’s Act states:

A child may consent to medical treatment if over 12 years and the child is of sufficient maturity and has the mental capacity to understand the benefits, risks, social and other implications of the treatment.^32^

The term ‘medical treatment’ is understood to be a manifestation of the right to health as provided for in Section 27 of the Constitution of the Republic of South Africa,^7^ and includes access to psychosocial and mental healthcare services.^33^

If an adolescent desires puberty blocking medication, HT or surgery, the IC process requires involvement of a multidisciplinary team, including both mental health and medical or surgical providers.^34^ It is recommended that both parents and legal guardians be included in this process wherever possible,^5^ as improved family support is associated with better mental health outcomes in TGD adolescents.^35,36^

### 2.4. Intellectual and developmental disability

Individuals living with intellectual or developmental disability have the right to access healthcare services.^37^ This includes TGD individuals who may have limited capacity to consent to gender-affirming treatment. Where fully IC cannot be provided, shared decision-making practices should be adopted, in which the client’s autonomy in the process is upheld.^38^

## 3. Primary care

### 3.1. The importance of the primary care provider

The TGD population is a marginalised group that faces many barriers in accessing healthcare services.^39^ Currently, there are few facilities, resources and targeted programmes to cater for this population’s specific sexual and reproductive health needs.^40^ In order to enable broader access, the provision of GAHC services needs to move away from specialist clinics and into primary care.^41,42^ Gender-affirming healthcare should be integrated into existing primary care services, as has been done with HIV care in South Africa. Primary care nurses are in a key position to ensure that TGD clients receive better care and experiences within healthcare facilities.^43^ Delivery of HT by primary care providers using the IC model can be performed safely and effectively for adult clients, with specialist endocrinologist care needed only for complex cases.^44^ Specialist involvement may also be of great value for an adolescent client; however, case-by-case decisions should be made within a multidisciplinary team context.^5^ In addition, a sex-positive approach by the primary care provider is important. It recognises that each individual’s sexuality is unique and multifaceted, and emphasises the importance of sexual pleasure, freedom and diversity.^45^

### 3.2. Comprehensive care

Screening is part of prevention and providing comprehensive primary care. When caring for a TGD client, specific attention needs to be paid to the following areas:

#### 3.2.1. Violence

Transgender and gender diverse persons experience a disproportionately high level of violence^39^ and, therefore, a trauma-informed primary care approach is essential.^46^ The World Health Organization (WHO) recommends the LIVES approach to violence (Listen, Enquire, Validate, Enhance safety and provide Support).^47^ A client who has experienced sexual violence needs timely access to appropriate care, including post-exposure prophylaxis (PEP), sexually transmitted infection (STI) prevention and, if necessary, emergency contraception.^48^

#### 3.2.2. Mental health

Comprehensive care should include screening for mental health conditions, as well as consideration of the possible negative impact of gender dysphoria on the client’s mental health, and the potential positive impact that gender-affirming treatment may have.^49,50^ In a South African study, it was found that transgender adults had an incidence of anxiety of 25.9%, of substance use 21.0%, of eating disorders or psychotic disorders 2.3%, and a lifetime prevalence of mood disorder of 21.2%.^51^ Assessment should include that of the client’s existing support structure, and support and psychoeducational needs related to their care.

A TGD client should always be offered mental health support,^5,6^ and continued support should be encouraged and facilitated, regardless of the client’s mental health status.^29^

A mental health condition is not a contra-indication to initiating HT, and it can be managed concurrently.^6^ Referral to a MHP is required if there is a concern about decision-making capacity or if a mental health condition needs to be addressed. Whilst the presence of some mental health disorders (particularly those with manic or psychotic features) may have an impact on an individual’s capacity to provide IC, a recent meta-review showed that most clients with a severe mental disorder made appropriate decisions regarding their healthcare.^52^ An Australian study^30^ revealed that general practitioners needed to refer only 8% of their TGD clients to a mental health professional prior to HT initiation and most of these clients had either schizophrenia or post-traumatic stress disorder (PTSD). Over half (56%) of TGD clients in this study had a mental health condition, such as depression, anxiety, attention-deficit-hyperactivity-disorder, autism-spectrum disorder or bipolar disorder; however, this did not have an impact on their capacity to consent to HT.^30^

#### 3.2.3. Substance use

Nearly half of transgender women (48%) and transgender men (49%) consume alcohol at hazardous, harmful or dependent levels.^39^ These harmful drinking practices are associated with a lifetime experience of physical or sexual violence.^39^ In addition, tobacco, alcohol and drug use can be used as coping mechanisms,^53^ and tobacco use in combination with oestrogen therapy is associated with an increased risk for venous thromboembolism;^5^ thus, screening is essential. A sensitive, client-centred approach within a harm reduction framework is recommended.^54^

#### 3.2.4. Fertility and contraception

Reliable contraception options must be explored in an assigned-female-at-birth (AFAB) client that has a uterus and ovaries, as pregnancy is still possible, even if the client is on testosterone.^55^

The client’s reproductive preferences should be thoroughly assessed, especially in the context of initiating HT. A transgender man who desires children may consider pregnancy^56^ and chest feeding.^57^ In a transgender woman who wishes to breastfeed, lactation can be induced by expression and medications (such as domperidone) with no adverse effects on the infant.^57,58^

#### 3.2.5. Cancer

Cancer screening is based on what anatomy (body part or organ) is present and whether the client meets the criteria for screening based on risk factors and/or symptoms. Relevant screening should be carried out regardless of HT use,^59^ and there is no evidence for increased risk of cancer as a result of HT.^59^

In a TGD client with cervical tissue, cervical screening, human papillomavirus (HPV) testing and HPV vaccination are essential.^60^ In South Africa, cervical cancer ranks as the highest cause of cancer-related deaths in persons AFAB^61^, and screening should be performed regardless of the sexual orientation or comorbidities.^62^ This can be performed with a Pap smear or a vaginal HPV swab test. A self-collected vaginal swab is an option for a client who is reluctant to have a vaginal examination.^63^

In a TGD client with breast tissue, recommendations for breast cancer screening should be followed as for a cisgender person.^64^

Prostate cancer has been documented in transgender women, although the prevalence is lower in transgender women than in cisgender men.^64^ Screening should follow guidelines as for cisgender men; however, if a prostatic-specific antigen (PSA) test is carried out in a transgender woman with a low testosterone level, the upper limit of normal should be reduced to 1.0 ng/mL (rather than 2.0 ng/mL as in cisgender men).^65^

#### 3.2.6. Sexual health

In a client taking feminising HT, changes to libido and sexual response cycle are usually observed within 1–3 months of initiation of treatment.^66^

In a client taking masculinising HT, an increase in sexual desire and activity is often reported^66^, and clitoral enlargement is likely to occur.^67^ Vaginal atrophy may occur because of the hypoestrogenic effect that testosterone has on vaginal tissues^68^ and can be ameliorated with lubricants.

A TGD client on HT may experience a shift in sexual orientation over time.^69^

#### 3.2.7. Sexually transmitted infections

Transgender and gender diverse clients are not a single category. Epidemiologic differences, such as the prevalence of gonorrhoea, require different responses for reducing infection and delivering appropriate sexual healthcare.^70^

A client may engage in high-risk behaviour, and a detailed sexual history should aid screening and examination. Assumptions about the client’s sexual orientation and behaviour should be avoided, and rather discussed in a -non-judgmental way. It is also important to note that in African culture, the thought of sex as taboo limits the range of acceptable terms when discussing a sexual history.^71^ The use of culturally respectful language can enable the reporting of truthful facts and minimise ambiguity or shame.^72^ This can be performed jointly with visual aids or a bilingual lexicon when necessary.^73^ Table 2^74^ provides recommendations for how to take a sexual history and the isiNguni alternatives provided in the table acknowledge respect and personhood-principles, that are largely characterised and embraced by Southern African ethno-cultural populations.

**TABLE 2:** Gender-inclusive language for taking a sexual history.

Common practice	Recommended practice	Suggested culturally sensitive options for South Africa
Addressing the client as Mr, Ms or Mrs	Call the client in from the waiting room using their last name only. Then ask them, ‘[h]ow would you like me to address you?’ (name and pronouns)	Pronouns are not gender related in Nguni. Titles include sisi/ bhuti/ mfowethu / mama/ baba / gogo / mkhulu. Addressing the client in a gendered manner is a form of respect for one’s age. The title loses the last letter when prefixing one’s name, for example, ‘sis’ Phumla’ Using the client’s Western name may not be respectful. Ask the client which name they would prefer to use as a sign of respect. Asking for and using the client’s clan name (Izithakazelo/iziduko) are gender neutral and respectful.
Use of binary sex markers (male or female)	Use a two-step gender question: ■ What is your gender? ■ What sex were you assigned at birth (i.e. is on your original birth certificate)?	Gender = ‘ isini /ubulili’ Ask ‘[w]hat was assigned on your birth certificate?’
Obtaining a menstrual or obstetric history	Avoid assumptions about anatomy. Clarify whether the client has a uterus.	A person that was assigned-female-at-birth will usually have a menstrual cycle. Ask ‘[do] you get your cycle?’ or ‘[d]o you often go on your cycle?’
Asking ‘[a]re you sexually active?’ or ‘[a]re you sexually active with men, women, or both?’	Advise the client that the questions asked might seem uncomfortable or intrusive but are intended towards assessing risk. Understand that there are many sexual practices that do not include penile-vaginal penetration. Therefore, rather ask, ‘[w]hat kinds of intercourse do you have?’, followed by appropriate questions regarding the specific sexual practices (e.g. penis-in-vagina, penis-in-anus, vulva-to-mouth, etc). Ensure the client guides the terminology used.	‘Sex’ is not usually a term used. Ask them ‘[i]n which ways do you and your partner make each other happy when you are together/ in the bedroom?’
Asking ‘[d]o you use condoms?’	Rather ask, ‘[d]o you use protection during sex?’ and ‘[w]hat protection do you use?’	Ask them ‘[d]o you use protection? Does your partner use protection when you are together?’

*Source:* Adapted from Stroumsa D, Wu JP. Welcoming transgender and nonbinary patients: Expanding the language of “women’s health”. Am J Obstet Gynecol. 2018;219(6):585.e5. https://doi.org/10.1016/j.ajog.2018.09.018

#### 3.2.8. HIV

Transgender and gender diverse persons are disproportionately burdened by HIV and have a greater risk of acquiring the virus, with the prevalence rate of HIV being 46% amongst transgender women in South Africa.^75^ As such, all TGD clients should be offered pre-exposure prophylaxis (PrEP).^76^ PrEP has no impact on the concentration of oestradiol or testosterone levels and can be safely prescribed in a client on HT.^77^ HIV testing and counselling services should address TGD-specific needs, and options, such as HIV self-screening, index testing and partner notification, should be offered.^59^

Modern antiretroviral treatment (ART) and the use of an integrase inhibitor are recommended for a TGD client with HIV, as there are no contraindications to HT.^78^ A dolutegravir-containing regimen is preferred over an efavirenz-containing regimen because it is generally better tolerated (fewer neuropsychiatric, hepatic and metabolic effects) and has a very high resistance barrier.^79^

If the TGD client is on both spironolactone and cotrimoxazole, serum electrolytes and renal function need to be frequently monitored because of a possible drug interaction, which may lead to hyperkalaemia, severe illness and even death.^80^ Particularly close attention should be paid to the client if they are elderly.^80^

Transgender women with HIV are less likely to access HIV treatment or engage in care because of barriers, such as poverty, violence, stigma and unemployment. As such, there are lower rates of virologic suppression and higher HIV-related mortality rates in this group.^81^

Adherence to ART and PrEP should be emphasised. Social media platforms and other information communication technologies should be used to encourage retention in HIV care services.^82^

### 3.2.9. Physical examination

It is important to note that a physical examination may cause the TGD client distress. Box 1 provides an affirming approach to a physical examination.^59^

BOX 1 An affirming approach to a physical examination.

- Adopt a trauma-informed approach, as many TGD clients find a physical examination uncomfortable or traumatic.
- Use correct pronouns and names. This is especially important in the context of a physical examination.
- Only conduct a genital examination if medically necessary.
- Explain to the client why the examination is necessary and what you will be doing.
- This can help to reduce anxiety. It is also an important part of obtaining informed consent.
- Be aware that the client may use alternative terminology for body parts - ask them which terms they would prefer you to use.
- Where possible, adapt procedures to make the client feel more comfortable (e.g. the client may be more comfortable with self-swabbing for HPV testing).

TGD, transgender and gender diverse; HPV, human papillomavirus.

## 4. Non-medical gender-affirming practices

It is important to understand non-medical practices and to establish which strategies the client may use. These strategies are used by TGD individuals to modify their gender presentation, and include binding, tucking, padding and packing.^83,84^ These strategies may alleviate gender dysphoria and can address the need to ‘pass’ as cisgender in a particular context.^84^ It is important to understand associated risks and benefits, and provide the client with information on how to perform them safely.

### 4.1. Binding

Chest binding is used to flatten chest tissue. Specialised compression garments, bandages or duct tape may be used. Although this can be safely performed, risks may include back and shoulder pain, shortness of breath, and skin and soft tissue problems. Recommend ‘off-days’ from binding, encourage good skin hygiene, and advise the client to avoid elastic bandages, duct tape and plastic wraps.^85^

### 4.2. Tucking

Tucking is used to present a flat pelvic area using a gaff (a specialised tight garment, often homemade), tape or tight briefs.^86^ The testicles are pushed into the inguinal canal, and the penis is taped between the legs.^87^ Although this can be safely carried out, risks may include testicular and penile pain, and skin problems such as a rash and itching.^86^ Recommend tucking for shorter periods or less tight tucking, and good skin hygiene is encouraged.^59^

### 4.3. Padding and packing

Padding involves the use of prosthetics or padding under the clothes to give the appearance of breasts and/or phenotypic female curves. Packing is the use of prosthetics or padding under the clothes to give the appearance of a penis and phenotypic male pelvic bulge.^83^ Both padding and packing carry little to no health risk.

## 5. Psychosocial care

The term ‘mental healthcare provider (MHP)’ has been used, and refers to the broad spectrum of providers who may assist the client with their psychosocial needs.^5,89^ These include clinical, counselling, educational and industrial psychologists; clinical, school and other social workers; psychiatrists; psychological and registered counsellors, and occupational therapists.

### 5.1. The role of the mental healthcare provider

A life-course approach alongside understanding the impact of minority stress, stigma and prejudice on the client’s psychosocial well-being is recommended.^2,88,89^ The concerns of the individual, as well as their broader socio-economic-cultural context, should be addressed. The term ‘mental healthcare provider (MHP)’ has been used, which refers to the broad spectrum of providers who may assist the client with their psychosocial needs.^5,89^ The MHP has many important roles in aiding gender-affirming care, as displayed in Table 3.

**TABLE 3:** The role of the mental healthcare provider.

Role	Description
Understand the complexities of ‘Assessment’ or ‘Evaluation’	The MHP needs to be cognisant that a mental health ‘assessment’ or ‘evaluation’ is particularly complex in relation to gender identity and gender-affirming healthcare in South Africa as it is a contentious concept that has historically been used to justify and maintain dominant ideology.^90^ The TGD client’s overall well-being and assessment or evaluation is a process of ‘coming to know and understand’ the client and their context.^91^ An asset-based approach should be used, and the focus should be on establishing and maintaining a sufficient support structure,^4^ thereby ensuring that the client understands the implications of gender-affirming medical interventions.
Aid diagnosis	Recognise any mental health difficulties that the client may be suffering from and develop interventions that centre the client and do not pathologise their gender identity. Depathologisation is evident in the ICD-11, as it conceptualises ‘gender incongruence’ as a ‘condition related to sexual health’,^92^ as opposed to the DSM-5, in which ‘gender dysphoria’ is classified as a mental health disorder.^93^
Provide support	Ensure that the client knows what to expect of planned interventions, help to develop strategies for strengthening their support system and support the client through any mental health challenges that may arise as a result of contextual responses to their gender identity. These may vary between children, adolescents and adults, and are affected by sociocultural and other factors.^5,6,94^
Provide psychotherapy	Provide supportive therapy, if desired by the client, before, during, and after social and physical transitioning. Intersectional challenges may increase the individual’s risk of experiencing minority stress, which then exacerbate the existing mental health disparities^21^ and impact negatively on continuity of care.^95^ Uphold best practice of care by ensuring understanding of and affirming the range of emotional, psychological and social outcomes that the client may experience, without imposing preconceived ideas.^1,6^ Mental health difficulties may result from contextual factors and environmental responses to the client’s TGD identity, individual genetic predisposition and non-gender identity-related causative factors.
Provide documentation	In terms of Act 49, the South African Department of Home Affairs requires two letters from medical professionals to enable a gender marker change.^96^ In addition, letters or reports may be requested by other healthcare providers involved in the client’s care. Respect the client’s autonomy and uphold confidentiality in all communication and write documents, as far as possible, in collaboration with the TGD client and other role players. The MHP should be cognisant of ethical considerations and act within the scope of practice.
Enable support groups	Facilitate TGD support groups for individuals and for the community. This will enable TGD individuals to access support and guidance through the gender-affirming healthcare process.
Advocate	Counteract stigma and violence, including hate victimisation in all its forms, across all developmental stages. Advocate for the TGD client’s human rights, and challenge inequality and oppressive systems that discriminate against the client.^6,21^

MHP, mental healthcare provider; TGD, transgender and gender diverse; ICD-11, International Classification of Diseases 11th revision; DSM-5, Diagnostic and Statistical Manual 5.

### 5.2. Children

A child can present as early as 2 or 3 years of age with persistent and consistent indicators of gender diversity.^5,94^ The MHP needs to ‘get to know’ the TGD child, and gender incongruence must be determined together with the child and their caregiver(s).^5,6,94^ Social transition is the recommended intervention for a TGD child, where it is their expressed need to do so^6^, and this can be facilitated by the MHP.^97^

### 5.3. Adolescents

The prospect of puberty and developing secondary sexual characteristics in conflict with experienced gender identity is often daunting and even traumatic for a TGD adolescent. The MHP should work with the adolescent and their caregiver(s) and, if appropriate, facilitate access to puberty pausing treatment.^5,6,94,98,99^

### 5.4. Adults

The MHP should offer an affirming and supportive space to enable TGD adults to come to an understanding and acceptance of their gender identity and its possible implications.^3,100^ Any trauma experienced as a consequence of the client’s gender identity should be addressed. A client who has begun transitioning within adulthood may require support with ‘coming out’ to their intimate partner(s), family, friends and work colleagues; and managing the resulting relational outcomes.^6,101,102^

For an elderly client who may have specific challenges, such as an increased risk of isolation or loneliness, the MHP should help to identify sources of strength and resilience.^103^ The MHP may need to assist in finding a safe and affirming living space with adequate medical and psychosocial care.

### 5.5. The meso and macro context

A MHP working with a TGD client (child, adolescent or adult) may need to engage with the client’s broader family, learning institution or community to help to establish safe and affirming spaces for the client. This could include supportive counselling, psychoeducation, community education, resource development and linkage, offering a support space and advocacy actions.^3,5,6,94^

## 6. Hormone therapy

### 6.1. Background

Gender-affirming hormones have been shown to be safe^104,105^ and effective,^11^ and were listed as essential medicines by the South African National Essential Medicine List Committee (NEMLC) in 2019, for tertiary level of care^106^. The goal of HT is to affirm the client’s experienced gender.^17^ In a non-binary client, it is particularly important to understand their desired outcome before deciding on treatment.^107^ Provision of HT should be based on the principle of IC, rather than on the specific diagnostic criteria that have previously, and often harmfully, been applied.^11^

### 6.2. Indications

In South Africa, the indications for accessing HT are as follows:

- A desire to use HT.
- Persistent gender incongruence between one’s experienced and assigned gender.
- Capacity to make a fully informed decision and consent to treatment.
- If the client is an adolescent, consult with a multidisciplinary team to confirm gender incongruence and mental capacity to provide IC.^108^
- If a significant medical or mental health concern is present, ensure that it is managed concurrently, without delaying HT.^30^
- Gender dysphoria and real-life experience (a period of time in which a TGD individual has lived full-time in their identified gender role) are not prerequisites for the initiation or maintenance of HT.^6^

Figure 1 shows a visual representation of the recommended process to follow when providing HT.

**FIGURE 1:**
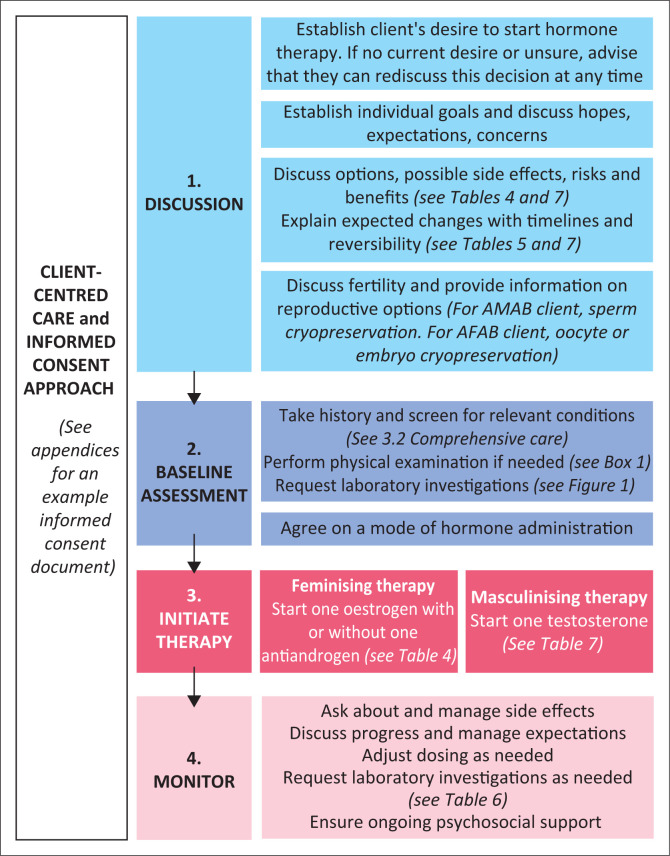
Recommended process for provision of hormone therapy.

### 6.3. Feminising therapy

The aim of therapy is to promote the development of feminising sexual characteristics and to suppress the masculinising effects of endogenous testosterone.^109^ The cornerstone of treatment is administration of exogenous oestrogen. The addition of an androgen receptor antagonist may be required to achieve full suppression of testosterone^110^; however, recent evidence suggests that this may not be essential to reduce testosterone levels to cisgender female ranges, as was previously thought.^111^ For conditions that may be exacerbated by oestrogen administration, such as oestrogen-sensitive malignancies, coronary artery disease and cerebrovascular disease, careful evaluation should be done prior to HT initiation^66^ and HT individualised. In a client with a history of venous thromboembolism (VTE), transdermal oestrogen may be considered after an IC discussion.^59,112^

Feminising treatment options are shown in Table 4, and effects and reversibility of treatment are shown in Table 5^126^. Baseline screening is recommended prior to HT treatment, as shown in Figure 2^127^.

**FIGURE 2:**
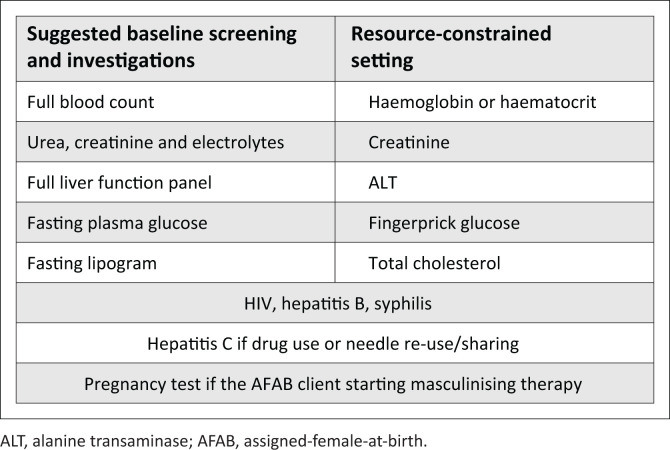
Suggested baseline screening prior to hormone therapy initiation.Baseline sex hormone levels are generally unnecessary.

**TABLE 4:** Feminising hormone therapy and antiandrogens.

Medication	Dose	Notes
**Feminising hormone therapy**		
Estradiol (patch) (Estradot)	Starting dose: 50 mcg – 100 mcg twice per week Increase by 100 mcg at a time Usual maintenance dose: 300 mcg – 400 mcg per week Maximum dose: 400 mcg per week	Safe and effective.^113^ Antiandrogen co-administration usually unnecessary. Use of multiple patches at a time may be impractical. Very low risk of venous thromboembolism (VTE).^113^ Possible side effects (rare): allergy to adhesive and skin irritation.
Estradiol valerate (IM or SC)	Starting dose: 6 mg once a week Increase by 2 mg at a time Usual maintenance dose: 6 mg – 10 mg per week (can consider dividing dose and giving every 3.5–5 days, rather than once a week) Maximum dose: 20 mg/week	Safe and effective.^108^ Antiandrogen coadministration usually unnecessary. Often a preferred treatment option.^114^ Very low risk of VTE.^108^ Possible side effects: migraine, nausea,^5^ mood changes, changes to libido and sexual response cycle.
17-beta-oestradiol (oral or sublingual) (Estrofem)	Starting dose: 2 mg daily Increase by 2 mg at a time Usual maintenance dose: 6 mg – 8 mg daily Maximum dose: 8 mg daily	Often requires antiandrogen coadministration. Not as safe as parenteral treatment^115,116^ Dose limitation because of associated risk of VTE.^117^ Possible side effects: migraine, nausea,^5^ mood changes, changes to libido and sexual response cycle.
Conjugated equine oestrogen (oral) (Premarin)	Starting dose: 0.625 mg – 1.25 mg daily Increase by 0.625 mg – 1.25 mg at a time daily Usual maintenance dose: 1.55 mg – 2.5 mg daily Maximum dose: 5 mg daily	Use only where bioidentical oestrogen (oestradiol) is not available. Often requires antiandrogen coadministration. Dose limitation because of moderate risk of VTE.^117^ Possible side effects: migraine, nausea,^5^ mood changes, changes to libido and sexual response cycle.
**Antiandrogens^66,118^**		
Spironolactone (oral)	Starting dose: 25 mg daily Increase by 25 mg at a time Usual maintenance dose: 50 mg Maximum dose: 200 mg	Risk of hyperkalemia: requires potassium monitoring. Use with caution if client on ACE-I/ARB. Possible side effects: diarrhoea, abdominal cramping, nausea, vomiting, headache, dizziness.
Cyproterone acetate (oral)	Starting dose: 10 mg–12.5 mg daily Increase by 5 mg–6.25 mg at a time Usual maintenance dose: 10 mg–25 mg daily Maximum dose: 25 mg daily	Potent antiandrogen, low doses should be sufficient.^119,120^ Possible side effects: sweating, agitation, fluid retention at high doses.
Bicalutamide^110,121,122,123,124,125^ (oral)	Starting dose: 25 mg twice weekly Increase by 25 mg twice weekly, or on alternating days Usual maintenance dose: 25 mg – 50 mg daily Maximum dose: 50 mg daily	Preferred antiandrogen as less risk of neurosteroid depletion (does not cross blood-brain-barrier readily). Possible side effects: constipation, back pain and fatigue.

IM, intramuscular injection; SC, subcutaneously; ACE-I, angiotensin-converting-enzyme inhibitor; ARB, angiotensin receptor blocker.

**TABLE 5:** Timeline and reversibility of feminising hormone therapy.

Effect	Time from initiation to onset	Time from initiation to maximum effect	Reversible
Body fat redistribution	3–6 months	2–3 years	Yes
Decreased muscle mass and strength	3–6 months	1–2 years	Yes
Skin softening	3–6 months	Unknown/variable	Yes
Change in sexual desire	1–3 months	3–6 months	Yes
Decreased erections	1–3 months	3–6 months	Yes
Breast growth	3–6 months	4 years	No
Decreased sperm production	Unknown/variable	> 3 years	Possibly
Decreased terminal hair growth	6–12 months	> 3 years	Yes
Scalp hair	Variable	Unknown/variable	Yes
Voice change	None	n/a	n/a

A client’s experience on treatment should be the primary guiding factor in dose titration and maintenance, and treatment may still be provided in resource-constrained settings where laboratory measurement of hormonal levels is not available. However, when these investigations are accessible, they can provide helpful guidance in optimising the dose. Recommended laboratory monitoring is shown in Table 6^59^.

**TABLE 6:** Laboratory monitoring for feminising and masculinising therapy.

Type of treatment	Investigation	Time since the initiation of treatment			
		1 month	3 months	6 months	Annually
Only if on feminising treatment	UEC or Cr + K^+^ (only if on spironolactone)	X	X	X	X
	Oestradiol (E2)†	X	X	X	
Only if on masculinising treatment	Haemoglobin/haematocrit	X	X	X	X
If on feminising OR masculinising treatment	ALT	X	X	X	X
	Total testosterone†	X	X	X	
	SHBG or free testosterone†	X	X	X	

Note: Compare results with the reference ranges consistent with the client’s gender.^59^

ALT, alanine transaminase; SHBG, sex hormone binding globulin.

† , Many laboratories will use references applicable to the client’s sex assigned at birth and not their gender.

### 6.4. Masculinising therapy

The goal of masculinising therapy is to promote the development of testosterone-induced secondary sexual characteristics.^128^ Suppression of oestrogen and ovulation will almost always occur^108^ and, thus, oestrogen antagonists are not required.

Exogenous testosterone can be administered by intramuscular or subcutaneous injection or as a topical transdermal preparation. Oral testosterone should be avoided as it is hepatotoxic.^67^ A client with severe hypertension, sleep apnoea or untreated polycythaemia (haematocrit above 55%) requires management prior to treatment initiation, as these conditions may be exacerbated by testosterone.^66^ Testosterone treatment options are shown in Table 7^66,129^, whilst effects and reversibility of treatment are shown in Table 8^66,109^. Baseline screening and monitoring are recommended, as indicated in Figure 2 and Table 6, respectively.

**TABLE 7:** Masculinising hormone therapy.

Medication	Dose	Notes
Testosterone cypionate 100 mg/mL (IM or SC)	Starting dose: 50 mg (0.5 mL) weekly Increase by 10 mg (0.1 mL) at a time Usual maintenance dose: 50 mg–80 mg (0.5 mL–0.8 mL) weekly or 100 mg–200 mg every 2 weeks Maximum dose: 100 mg (1 mL) weekly or 200 mg every 2 weeks	More affordable than long-acting injection. Avoid in pregnancy.^128^ Possible side effects: polycythaemia, acne, androgenic alopecia, amenorrhea, loss of fertility, mood changes, dyslipidaemia and hypertension. Take sample for testosterone measurement at peak, halfway between doses, target the safe upper limit of reference range.
Testosterone undecanoate (IM) (Nebido)	Starting dose: 1000 mg; given every 10–12 weeks Increasing the frequency, rather than raising the dose is required Usual maintenance dose: 1000 mg every 10–12 weeks Maximum dose: 1000 mg	More expensive than the short-acting injection. Avoid in pregnancy^128^ Possible side effects: polycythaemia, acne, androgenic alopecia, amenorrhea, loss of fertility, mood changes, dyslipidaemia and hypertension. Achieving the correct dose can be difficult with long dosing intervals. Take sample for testosterone measurement at trough, target the lower limit of reference range.
Topical testosterone (Androgel)	Starting dose: 1 sachet (5 mL) daily topically Increase by 1 mL at a time Usual maintenance dose: varies by client Maximum dose: limited by body surface for application	Only available from compounding pharmacies. Avoid in pregnancy.^128^ Possible side effects: polycythaemia, acne, androgenic alopecia, amenorrhea, loss of fertility, mood changes, dyslipidaemia and hypertension.

IM, intramuscularly; SCI, subcutaneously.

**TABLE 8:** Timeline and reversibility of masculinising hormone therapy.

Effect	Time from initiation to onset	Time from initiation to maximum effect	Reversible
Skin oiliness and acne	1–6 months	1–2 years	Yes
Facial and body hair growth	6–12 months	4–5 years	No
Scalp hair loss	6–12 months	Unknown	No
Increased muscle mass and strength	6–12 months	2–5 years	Yes
Fat redistribution	1–6 months	2–5 years	Yes
Cessation of menses	2–6 months	n/a	Possibly
Clitoral hypertrophy	3–6 months	1–2 years	No
Vaginal atrophy	3–6 months	1–2 years	Yes
Deepening of voice	6–12 months	1–2 years	No

### 6.5. Adolescents

Whilst HT is not required for prepubertal TGD children, pubertal suppression to halt the progression of physical changes may significantly reduce distress in a TGD adolescent,^94,108^ which, in turn, has been shown to improve mental health conditions and decrease suicidality.^130^ Puberty can be suppressed with gonadotrophin-releasing hormone agonists (GnRHa) once Tanner Stage 2 of puberty has been reached.^94^ Gonadotrophin-releasing hormone agonists available in South Africa include leuprolide and goserelin, both of which are administered every 12 weeks via intramuscular or subcutaneous injection.^131^ It is recommended that a paediatric endocrinologist oversees this care,^5,108^ and that fertility preservation is discussed prior to HT initiation.^132^ The timing of HT initiation should be individualised, and should consider family support, likely time on GnRHa, potential impacts on height, risks of delaying HT and the adolescent’s ability to consent.^94^ The inclusion of an MHP and, ideally, the parents or legal guardians are recommended when deciding on the appropriateness of HT.^5^

### 6.6. Mature clients

Hormone therapy is indicated as a long term treatment, as some body changes may reverse if it is stopped.^59^ There is no age recommendation for the reduction or termination of HT, and individual cardiovascular risk in the mature TGD client needs to be considered and discussed with the client.^59^

## 7. Surgery

### 7.1. Preoperative considerations

It is important to note that there is diversity in the surgery requested by TGD clients.^133^ A client may desire for chest or facial or genital surgery only, or a combination of these. A non-binary client’s request for surgery should be specifically individualised.^134^ Tables 9 and 10 show, respectively, the available feminising and masculinising surgical options. Hormone therapy is usually recommended prior to surgery; however, a client may be unable to or prefer not to take HT prior to surgery.^6^ In South Africa, a documented process of thorough IC is essential prior to surgery.

**TABLE 9:** Feminising surgery.

Type	Before surgery	Notes
**Breast surgery** Insertion of silicone breast implants if larger breasts are desired^134^	Recommend 1 year of prior HT for the best outcome (maximum breast development occurs after 3–5 years)^6^	Two weeks of postoperative antibiotics are provided routinely. Breast implants carry a lifelong risk of infection^135^
**Facial feminisation** Tracheal shave is the commonest procedure^134^	No requirement for HT prior to surgery	Consider facial contouring using hyaluronic acid fillers as an alternative to surgery^136^
**Orchidectomy** Removal of testes	Recommend 1 year of prior HT to provide gradual transition from testosterone; this is physiologically much safer Discuss reproductive options (e.g. sperm cryopreservation)	Haematoma is the commonest complication
**Genital surgery^137^** (to create a vagina) Penile inversion vaginoplasty or Colonic interposition vaginoplasty	Recommend 1 year of prior HT Advise pubic hair removal with electrolysis or laser^137^ Foreskin stretching is required for penile inversion	Penile inversion carries less risk of scarring and long-term closure^137^ Shortening of the urethra may lead to urethral stricture^137^ Colonic interposition vaginoplasty carries the risks of abdominal surgery and permanent vaginal mucus discharge

HT, hormone therapy.

**TABLE 10:** Masculinising surgery.

Type	Before surgery	Notes
**Breast surgery with chest reconstruction^134^** Mastectomy Areola and nipples often require size reduction^138^ Most desired operation for most transgender men^134^	No requirement for HT prior to surgery^6^ Prior chest binding technique may affect outcome because of reduced skin elasticity^139^	Surgical technique depends on the size of breasts and quality of tissues^138^ Commonest complication is seroma formation; therefore, a drain is usually left in for 5–10 days^138^
**Genital surgery** (to create a penis) **Radial forearm phalloplasty** Permits standing to urinate but not erection^137^	Recommend 1 year of prior HT^6^ Advise donor site hair removal with electrolysis (to prevent hair inside urethra)	Commonest complication is urethral fistula or stricture. About 50% of clients will require a secondary operation^137^
**Metoidioplasty** Conversion of clitoris into a penis,^137^ has the benefits of erection and erogenous sensation	Recommend 1 year of prior HT^6^ Usually requires prior enlargement of clitoris using topical testosterone cream and a pump for tissue expansion^137^	Penis may be too small for penetrative sex without pre-operative preparation^137^ Commonest complication includes urethral fistula or stricture. About 25% of clients will require a secondary operation^137^
**Hysterectomy** Removal of uterus	Recommend 1 year of prior HT^6^ Discuss reproductive options (oocyte cryopreservation)	Carries complications of abdominal surgery

HT, hormone therapy.

### 7.2. Peri-surgical care

Post-surgical care is vital to recovery, and should include psychological care and physiotherapy. It is important to note that the continuity of gender-affirming care does not end with the surgical procedure(s), and ongoing support should be provided. The TGD community can play a significant role in perioperative care, both through peer and organisational support groups.^140^ Satisfaction following surgery is usually high, with less gender dysphoria, reduced psychological distress and better integration into society.^141^

## 8. Institutions

### 8.1. Care facilities

Providing a safe, welcoming, and culturally appropriate healthcare environment is essential to ensure that a TGD client not only seeks care but also returns for follow-up.^59^

The following are recommendations for care facilities, including healthcare facilities,^142,143^ old-age homes^103^ and shelters^144^:

- Ensure that staff are trained to care for a TGD client, and that anti-discrimination and anti-harassment policies are in place.
- Limit language as a barrier by ensuring that there is staff competency to present information in more than one of South Africa’s 11 official languages and, if required, basic South African sign language.
- Ensure that the client’s gender identity and treatment information are kept confidential and protected under the Protection of Personal Information Act (POPIA).
- Ensure registration records and intake forms reflect the client’s name-in-use, legal name and surname (if relevant and in consultation with the client), pronouns and gender.
- Practise discretion with billing information in terms of differentiating between the client’s legal name and name-in-use, and consult with the client directly to avoid any breaches of confidentiality.
- Respect a person’s name and pronouns, regardless of the appearance, history or sex assigned at birth.
- Assign the person to a bed or room or ward based on their self-identified gender.
- Ensure the client’s equal and fair access to bathroom facilities that are aligned with their self-identified gender (including fully private, non-binary or gender-neutral bathrooms).
- Ensure that the client has access to personal items that facilitate their gender expression (this may include items, such as makeup and shaving equipment, and items used to bind, pack or tuck).
- Ensure residents in shelters are able to choose their clothing, residential allocation (e.g. in single-gender settings)^144^ and are protected from gender-identity discrimination.^103^

### 8.2. Correctional facilities

The following are specific recommendations for TGD offenders:

- Ensure that all correctional staff are trained on gender identity and diversity, and that all in-house health providers are trained in GAHC.^145^
- Ensure safe and secure detention and incarceration, with appropriate section placement to reduce victimisation.^146^
- Assign the offender to a single cell, if this is their preference, but recognise that this protective placement might in itself result in victimisation.^147^
- Facilitate access to HT.^148^
- Ensure that the offender has access to sexual healthcare (provision of condoms, PEP and PrEP), given their increased exposure to HIV and STIs.^146^

### 8.3. Work facilities

Inclusion begins before a TGD staff member’s social transition within the workplace. Collaboration between leadership and human resources is needed for the implementation of clear guidance to support TGD staff.^149^ Healthcare providers can play an advisory role in needs assessment, intervention design and implementation, and policy development and employee benefits.

### 8.4. Educational facilities

In accordance with South African legislation, all schools, whether private or public, mixed or single gender, must ensure an inclusive, non-discriminatory and diversity-affirming environment.^150,151,152^ This supports basic human rights, actualisation of potential, human dignity, equality, right to education, protection from physical and emotional harm, and is in the best interest of the learners. Healthcare providers can assist schools with the development of relevant policies and guidelines, as well as staff sensitisation.^3^

## 9. Voice and communication

It is helpful to understand how sex and gender influence voice and communication, and that a speech-language therapist (SLT) can play an important role in this regard.^6^ Voice and communication are often closely connected to gender identity or expression, and the TGD client may want to sound more feminine, more masculine or gender neutral.

Masculinising HT can contribute to a desired voice change but may not be sufficient to achieve the client’s goals. Feminising HT is unlikely to result in a desired voice change. The TGD client, therefore, may benefit from referral to a qualified SLT with experience in providing gender-affirming care.^6^ The main strategy for voice care is related to the alteration of one’s speaking fundamental frequency, intonation and resonance.^153,154^

The SLT should perform a voice and communication assessment, which includes a quality of life measure,^155,156^ and can provide both voice and communication interventions, as shown in Table 11. It is important to acknowledge South Africa’s multilingual communication landscape, and that communication requires an individualised approach and specialist intervention.

**TABLE 11:** Voice and communication interventions.

Voice	Communication
Vocal stretches and voice conditioning Increased or decreased speaking frequency and/or resonance Perceptual-motor learning, for example targeting sensations or images^157^	Pragmatic aspects, including conversation, discourse and alternation Non-verbal communication, including facial expressions and tactile communication

## 10. Key terms

Table 12 shows important key terms used within the field of GAHC.

**TABLE 12:** Key terms.

Term	Explanation
Cisgender	Abbreviated as ‘cis’, describes a person whose gender identity and expression match their sex assigned at birth.^3^
Gender	‘An institutionalised system of social practices for constituting people as two significantly different categories, men and women; and organising social relations of inequality on the basis of that difference’.^158^ Gender is based on social norms and expectations. In many cultures, people are divided into a gender binary of either men or women; however, there are also cultures that recognise other genders, sometimes as a third-gender category, or as a range of non-binary identities, for example genderqueer, gender fluid, and also as bigender or agender. Most societies have a history of systemic gender inequality, with men occupying a privileged position and women being subjected to socio-economic disadvantage, discrimination and violence. Because of colonisation, the binary Western perspective has been entrenched within our society.
Gender dysphoria	The psychological and/or physical distress caused by the incongruence between sex assigned at birth and gender identity. Not all TGD individuals experience gender dysphoria; however it can be debilitating for some. Although gender dysphoria is a medical diagnostic classification in the Diagnostic and Statistical Manual-5 (DSM-5).^93^ TGD individuals’ experiences of it are diverse and may affect their lives in various ways.
Gender expression	Aspects of a person’s physical appearance and behaviour, which is defined culturally or socially to be either masculine or feminine. Every society has its own normative assumptions and prescriptions about how women and men should feel, dress, act and work. Gender expression can also be fluid or non-conforming.^159^
Gender identity	Defined by the Yogyakarta principles (South Africa is a signatory to these principles) as ‘each person’s deeply felt internal and individual experience of gender, which may or may not correspond with the sex assigned at birth, including the personal sense of the body (which may involve, if freely chosen, modification of bodily appearance or function by medical, surgical or other means) and other expressions of gender, including dress, speech and mannerisms’.^9^
Cis-heteronormativity	Refers to the hierarchical system of power, prejudice and discrimination, in which cisgender and heterosexual individuals are privileged above sexual and gender diverse (or perceived sexual and gender diverse) persons.
Intersex	Refers to persons born with sex characteristics, such as chromosomes, gonads and genitals, which do not fit typical binary notions of male or female bodies. Intersex is an umbrella term used to describe a wide range of natural bodily variations.^160^ Some people with intersex traits self-identify as intersex, and some do not. Some prefer the term *Differences of Sex Development* or *Diversity of Sex Development* (*DSD*). The medical term Disorder of Sex Development is often considered derogatory by intersex persons as difference or diversity should not automatically be pathologised.
LGBTQIA+	An umbrella term for communities who, for different reasons, have a shared experience of marginalisation and discrimination in society, and who have shared goals of improving access to human rights and basic freedoms. L stands for lesbian, G for gay, B for bisexual, T for transgender, Q for queer or questioning, I for intersex, A for asexual or agender, and + indicates developing language and the inclusion of other diverse gender identities and sexual orientations.
Misgendering	Intentionally or unintentionally using an inaccurate pronoun or description in a way that undermines a person’s gender identity. Similarly, *deadnaming* (*necronym*) refers to using a TGD person’s previous given name, despite them having changed their name, or asked to be addressed by a name that reflects their gender identity.
Non-binary	A range of gender identities that do not fall into the traditional binary categories of male or female. It is important to recognise that this gender binary does not describe the identity of many people. People with non-binary gender identities may identify as gender fluid, gender diverse, agender, genderqueer, gender non-conforming, transmasculine, transfeminine or various other non-binary identities.
Sex	A complex interplay of multiple physical characteristics (including hormones, internal reproductive organs, gonadal tissue, genitalia and chromosomes) that cannot be categorised into a binary of male or female. When a child is born, they are usually assigned as either female child (assigned female at birth [AFAB]) or male child (assigned male at birth [AMAB]) based solely on the observed external genitalia at birth. This does not account for intersex individuals or for diversity of sex development (DSD), which is problematic.
Sexual orientation	Describes who one is intimately attracted to, and with whom one has emotional or sexual relationships and the sexuality one may identify with. *Sexual orientation is not the same as gender identity.* Gender identity refers to a person’s experience of their own gender, and sexual orientation refers to their attraction to others. A person’s gender identity does not in any way predict their sexual orientation.
Transgender	A term that describes a person who does not identify (wholly or partially) with their sex assigned at birth. A *transgender woman* is someone who was assigned male at birth (AMAB) but who identifies as a woman. The previous term MTF (male-to-female) is no longer considered widely acceptable or accurate. A *transgender man* is someone who was AFAB but who identifies as a man. The previous term FTM (female-to-male) is no longer considered widely acceptable or accurate.
Transphobia	An irrational and systemic hostility towards people who are transgender, gender diverse, or who otherwise do not fall into traditional gender categories and norms.

TGD, transgender and gender diverse.

## Figures and Tables

**FIGURE 1-A1:**
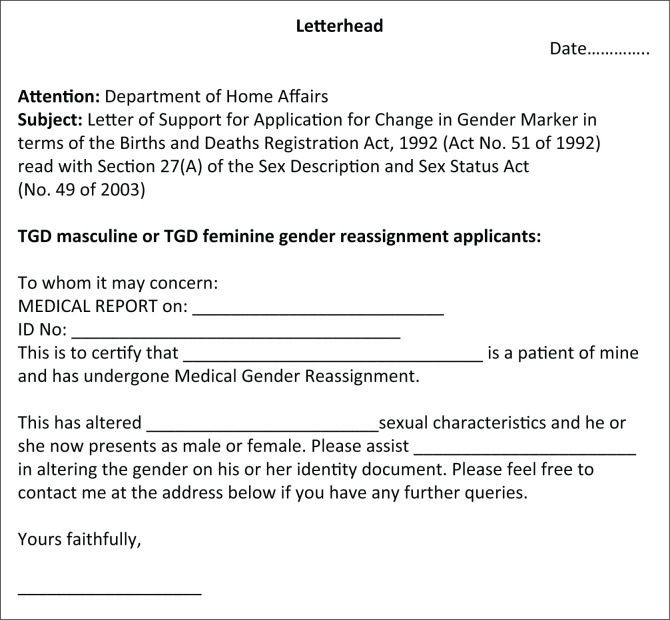
Suggested letter in support of application to Department of Home Affairs for change in gender marker – Medical doctor.

**FIGURE 2-A1:**
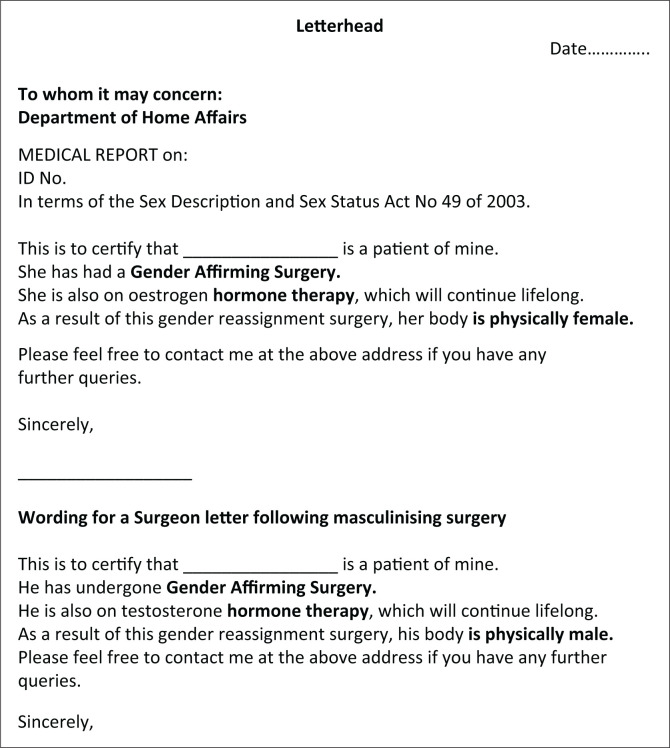
Suggested letter in support of application to Department of Home Affairs for change in gender marker – surgeon (following feminising surgery).

**FIGURE 3-A1:**
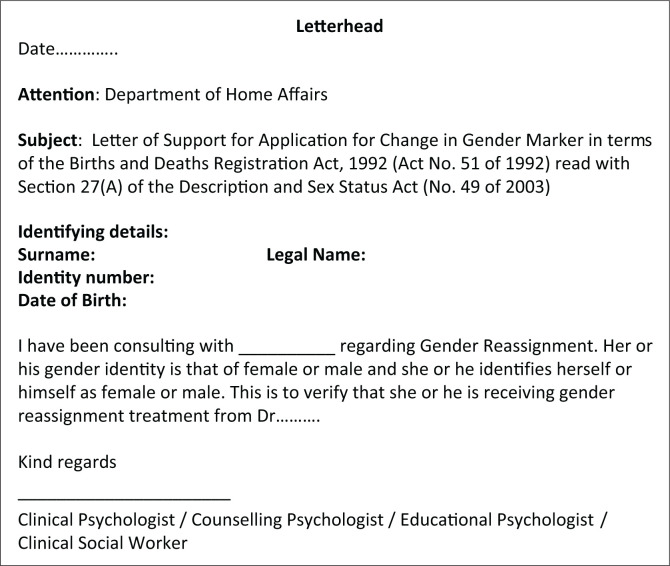
Suggested letter in support of application to Department of Home Affairs for change in gender marker – clinical psychologist or counselling psychologist or educational psychologist or clinical social worker.

## Appendix 1: The role of health professionals in change of gender marker at Home Affairs: Act 49

South African legislation allows a transgender and gender diverse (TGD) person to change his or her gender marker, as well as his or her forename. Transgender and gender diverse people can apply to change their sex description in the birth register in terms of the *Alteration of Sex Description and Sex Status Act* 49 of 2003. Section 2(1) states:

Any person whose sexual characteristics have been altered by surgical or medical treatment … resulting in gender reassignment … may apply to the Director-General of the National Department of Home Affairs for the alteration of the sex description on his or her birth register.^96^

In terms of section 2(2)(b), an application must include two reports by medical practitioners, the examples of which are provided below. The Act does not make gender reassignment surgery compulsory. Hormonal treatment is sufficient.^96^

Application for forename change can be made as per Section 24 of the Births and Deaths Registration Act and does not require any letter.^161^

A helpful resource that details the process for clients is available at https://www.betrue2me.org/resources/be-true-2-me-guideline-legal-gender-marker-and-forename-change/

## Appendix 2: Client information and consent form for feminising hormone therapy

The informed consent forms contained herein are included as examples of what such a document might look like, and the kind of information it may contain. These are not intended to be used ‘as is’ but should rather serve as a template or guideline to practitioners to help them craft their own informed consent forms, specific to their practices.

Many TGD clients choose to seek gender-affirming care in the form of hormone therapy (HT). The decision to start on HT rests with you, the client, and not with your healthcare provider. The informed consent model of treatment provides you agency over this decision – it is the role of your doctor to support and guide you through this process **safely** and **effectively**.

You may have read up, or heard from other TGD people, about HT. Some of the information that you may have come across could potentially be out of date or inaccurate.

If you have any questions or concerns at any time, you should always **feel free** to **raise these concerns** with your healthcare provider.

Please remember that **every client is unique** and will respond differently to medication, and that one client’s treatment programme might differ substantially from another because of a variety of physiological and medical factors. **Try not to compare** the treatment you are receiving with that of anyone else – your journey is your own. If you have concerns about the efficacy of your treatment, make a point to discuss this with your provider at your next appointment.

Before starting on HT, there are a few points that are worth considering. This informed consent document will draw your attention to some of these points, as well as outline the expected effects, side effects and risks that are associated with HT to make sure that you have all the information you need to make the best decision about your body and your health.

### The role of psychotherapy in transgender and gender diverse clients

Visiting a psychologist **is not a requirement** for initiating HT. Whilst previously some healthcare providers required a letter of diagnosis or referral, this is no longer necessary under international best practice.

Your doctor will ask you some questions to determine that you have a **good support structure** as you move forward. This is not because going on HT itself necessitates this but rather because for many clients of TGD experience, navigating the world is already difficult, and HT, although often resulting in **many positive and beneficial changes**, can also lead to some **emotional lability**. A solid support structure looks different for everyone; however, this may include friends, families, support groups, therapists or counsellors. Your doctor might suggest or offer you referrals to support groups and therapists, if you indicate that you might benefit from these.

### The role of endocrinologists and other specialists

Hormone therapy **does not need** to be prescribed or monitored by an endocrinologist. Prescribing HT is well within the realm of a suitably skilled general practitioner (**GP**) **or family physician**. Not all GPs, nor all endocrinologists, have experience in managing gender-affirming HT, and the expertise of the clinician should be the guiding factor in determining who prescribes and monitors your HT.

You may benefit from visiting other **allied healthcare professionals**, such as **speech therapists**, or from medical specialists, such as **plastic surgeons**. Not every TGD client will necessarily want to pursue these options, and you should discuss your individual goals with your prescribing doctor.

### Home affairs and gender marker

If you wish to update your gender marker on your birth certificate and ID document, the Department of Home Affairs requires **two letters from healthcare professionals,** which state that you have undergone **medical or surgical** gender reassignment. Either of these is sufficient; you do **not need to have had surgery** to update your gender marker. Unfortunately, at present, gender markers in South Africa are binary; thus, an ID document can reflect either female or male gender; there is no unspecified or non-binary marker. Your prescribing doctor can write one of your letters for the Department of Home Affairs and should be able to refer you to another healthcare provider to write the second letter.

If you wish to change your name, this process should be undertaken separate to updating your gender marker (either before or after). This **does not require supporting letters** from healthcare providers.

Other important aspects to explore:

**Potential challenges with legal documents:** Some TGD persons have trouble with banking, registration as a student and writing examinations, registration of motor vehicles, and so on whilst they are waiting for their new documents. Some are accused of potential fraud because they do not look the same as the photograph in their identity document or their name may be different from the name on their qualification certificates. Changing one’s gender marker and names will take a while. You may want to consider how you will deal with it in the interim. You also may want to consider whether you need to change other documentation, for example your matric certificate.

**Potential impact on emotions:** The impact of hormones can be very diverse and individualised. Your mood may fluctuate. For example, some TGD women may experience being more moody or tearful at times.

**Potential impact on relationships with family and significant others:** Have you thought about the possible impact on your relationship with significant others? If you are in an intimate relationship, this may change when you start on hormones, and relationship roles may need to be renegotiated. A partner may grieve the loss of aspects of who you were and the way the relationship used to be. What possible impacts can it have on your family and how will you be able to deal with your family’s response? Have you considered the impact of the change of gender role in your family? Have you considered the impact of potential loss of fertility? Are there children that may be impacted and are the children prepared?

**Potential change in sexual orientation:** It is possible that your sexual orientation may remain constant or shift, either temporarily or permanently (e.g. shift in attraction or choice of sexual partners, widened spectrum of attraction and shift in sexual orientation identity).

**Potential impact on safety:** In some settings, the physical changes in hormones may have an impact on your safety, with people who do not fit into society’s expectations of male or female being at increased risk of violence.

**Potential impact on employment:** Some TGD persons experience discrimination in the workplace or struggle to obtain employment. This may be more difficult when your legal documents (e.g. identity document) and your appearance do not match.

**Potential grief and loss:** Some TGD people experience a sense of loss, for example a TGD woman may be treated differently by society when she is read as a female person. A TGD woman may also experience that she has become physically weaker.

**Taste changes:** Some TGD persons experience a change in their taste sensations, and their likes and dislikes of certain foods.

**Body odour change:** Some TGD persons experience a change in their body odour on hormone treatment.

**Appetite and sleeping patterns:** Often the TGD person will experience an increase of appetite, which, in turn, could lead to weight gain. Sleeping patterns may also be affected.

### Feminising hormone therapy

Feminising HT is prescribed for assigned-male-at-birth clients who wish to feminise.

The backbone of feminising HT is oestrogen therapy. Anti-androgens (spironolactone, cyproterone acetate or bicalutamide) are sometimes used, although many clients can achieve testosterone suppression using oestrogen therapy alone.

The biggest concern with oestrogen therapy is the risk of clot formation, which can lead to deep venous thrombosis (DVT), or life-threatening pulmonary emboli. It is this risk that limits the amount of oestrogen we can safely give clients. Oral oestrogen (e.g. Estrofem or Premarin) carry a higher risk of these adverse events than parenteral (i.e. administered outside the digestive tract) oestrogen.

**Table:** Comparison of oestrogen preparations.

Type	Examples	Advantages	Disadvantages
Tablet, taken orally	Estrofem Premarin	Easy to use Accessible at most pharmacies	Higher thrombotic risk
Tablet, dissolved sublingually†	Estrofem	Accessible at most pharmacies	Time consuming (takes 30 min to absorb)
Injectable	Estradiol valerate	Safer Affordable Once-a-week dosing	Available only from compounding pharmacy Requires knowledge of injection technique Requires disposables (syringes, needles and alcohol swabs)
Patch	Estradot	Safer Available at most pharmacies Twice-a-week dosing	Patches need to be applied and cared for correctly May cause skin reactions because of adhesive.

† , Taking an oestrogen tablet sublingually involves holding the tablet in the mouth – either under the tongue, inside the cheek, or between the lips and teeth, whilst the tablet dissolves. After dissolving, the residue must be held in the mouth for a further 30 min to fully absorb, and any remnants need to be spat out and the mouth thoroughly rinsed. It is important not to swallow at all during this time, as any swallowed medication will move through the digestive tract and carry a higher risk of adverse events.

Your doctor will discuss with you the various options for HT that are available and help you to decide which form of treatment is best for you.

### Costs of hormone therapy

It is important to remember that not only do different clients have different needs in accessing HT but also that prices for medication may vary between different pharmacies, and that these prices may fluctuate over time. The majority of clients can expect to spend approximately R300–R500 per month on HT. This does not include monitoring blood tests or doctor’s visits.

### Changes that occur when using hormone therapy

The changes you will experience on HT often take some time to fully develop. Some of these changes are reversible, and will disappear should you discontinue HT. Others are irreversible and will persist even if you stop taking your hormones.

The timeline for these changes to begin is variable; however, most of them will only reach their maximum degree after 3–5 years on HT.

#### Reversible changes

- loss of muscle mass and decreased strength
- changes in body fat distribution, possibly associated with weight gain (increased fat deposition in breasts, buttocks, hips and thighs)
- softer and thinner skin
- reduced acne
- lighter and thinner body and facial hair
- cessation of male-pattern balding, possible scalp hair regrowth
- changes in sex drive (usually a decrease initially, followed by an increase together with a change in sexual response cycle)
- changes in the strength and frequency of erections, and changes in the amount and consistency of ejaculate
- changes in mood and emotional response.

#### Irreversible changes

- breast development; whilst the size of breast tissue may fluctuate, HT will cause permanent development of breast structures, which will remain even if HT is withdrawn
- testicular atrophy
- infertility
- changes in bone density.

### Limitations of hormone therapy

It is important to understand that there are certain features that HT cannot alter, which include the following:

- presence of facial hair – although HT may make the hair thinner, or cause it go grow more slowly, HT will not eliminate facial hair
- pitch of the voice
- bone structure of the face
- presence of thyroid cartilage (Adam’s apple).

### Important risks associated with hormone therapy

As with any medication, HT carries with it certain risks. Some of these risks can be mitigated or reduced by lifestyle factors, whilst others are independent risks that cannot be altered. It is important to be fully aware of the risks associated with HT before starting your treatment.

Blood clots are the most prominent risk factor associated with feminising HT. A blood clot can lead to DVT, pulmonary embolism (a blood clot in the lungs), heart attacks or strokes. These conditions may be severely debilitating or even fatal:

- cardiovascular disease
- nausea or vomiting
- migraines or other headaches
- gallstones and other diseases of the gallbladder.

Elevated levels of prolactin can rarely occur in clients on feminising HT because of the development of a prolactinoma, a benign (non-cancerous) tumour of the pituitary gland, which may interfere with vision. These can require surgical management, depending on the nature of the lesion.

Some of the risks mentioned are modified by other factors. Notably, cardiovascular and clot risk are worse in clients who

- are above the age of 45
- smoke
- use alcohol
- have pre-existing medical conditions, such as diabetes, high blood pressure and high cholesterol.

Some clients will experience a reduction in their blood pressure and improvements in their cholesterol levels on HT. This is not a guarantee and is not a replacement for positive lifestyle changes.

### Fertility

Although not all clients become infertile on HT, and some might regain fertility if they stop HT, many may become irreversibly infertile. Hormone therapy is not a replacement for effective and responsible contraceptive use.

All clients considering starting on HT should consider using a Cryobank to preserve genetic material, in case they wish to conceive genetically related children at a later stage. Even if this is not a priority for you at this stage in your life, please consider the possibility that your perspectives might change with time, and that it is ideal to store material before starting HT rather than trying to regain fertility once you are already on hormone treatment.

Your doctor can refer you to facilities that can assist in cryopreservation.

### Monitoring and follow up

Your doctor will advise and guide you in monitoring your safety while you are on HT. Usually, this will involve regular check-ups and physical examinations, as well as certain blood tests.

At the outset, it is not uncommon for these evaluations to be performed monthly, whilst you are still achieving the correct hormonal balance for you. Later, once you are stable on treatment, these intervals might be extended to 6-monthly, or perhaps even annually. This schedule is different for every client.

If you decide to stop your HT, you should discuss this decision with your doctor. It can be dangerous to abruptly withdraw HT without adequate medical supervision.

### More information

Please remember that you can discuss any questions or concerns with your doctor at any time.

Information on self-injection technique can be found at: https://fenwayhealth.org/wp-content/uploads/2015/07/COM-1880-TGD-health_injection-guide_small_v2.pdf

### Informed consent for feminising hormone treatment

I confirm that I have read and understand the information above.

I confirm that my doctor has told me about the effects of feminising hormone treatment,

including the more common or serious risks and side effects as mentioned above.

I understand that some of these effects may be permanent.

I understand that as part of my treatment plan, I shall take my medication as prescribed and have check-ups, including blood tests as required.

My doctor has offered me adequate opportunity to ask any questions that I have regarding feminising hormone therapy.

I hereby agree that my doctor starts/continues treating me with feminising hormone therapy.

## Appendix 3: Client information and consent form for masculinising hormone therapy

The informed consent forms contained herein are included as examples of what such a document might look like, and the kind of information it may contain. These are not intended to be used ‘as is’ but should rather serve as a template or guideline to practitioners to help them craft their own informed consent forms, specific to their practices.

Many TGD clients choose to seek gender-affirming care in the form of HT. The decision to start on HT rests with you, the client, and not with your healthcare provider. The informed consent model of treatment provides you agency over this decision – it is the role of your doctor to support and guide you through this process **safely** and **effectively**.

You may have read up, or heard from other TGD individuals, about HT. Some of the information that you may have come across could potentially be out of date or inaccurate.

If you have any questions or concerns at any time, you should always **feel free** to **raise these concerns** with your healthcare provider.

Please remember that **every client is unique** and will respond differently to medication, and that one client’s treatment programme might differ substantially from another’s because of a variety of physiological and medical factors. **Try not to compare** the treatment you are receiving with that of anyone else – your journey is your own. If you have concerns about the efficacy of your treatment, make a point to discuss this with your provider at your next appointment.

Before starting on HT, there are a few points that are worth considering. This informed consent document will draw your attention to some of these points, as well as outline the expected effects, side effects and risks that are associated with HT to make sure that you have all the information you need to make the best decision about your body and your health.

### The role of psychotherapy in transgender and gender diverse clients

Visiting a psychologist **is not a requirement** for initiating HT. Whilst previously some healthcare providers required a letter of diagnosis or referral, this is no longer necessary under international best practice.

Your doctor will ask you some questions to determine that you have a **good support structure** as you move forward. This is not because going on HT itself necessitates this, but rather because for many clients of TGD experience, navigating the world is already difficult, and HT, although often resulting in **many positive and beneficial changes**, can also lead to some **emotional lability**. A solid support structure looks different for everyone; however, this may include friends, families, support groups, therapists or counsellors. Your doctor might suggest or offer you referrals to support groups and therapists, if you indicate that you might benefit from these.

### The role of endocrinologists and other specialists

Hormone therapy **does not need** to be prescribed or monitored by an endocrinologist. Prescribing HT is well within the realm of a suitably skilled **GP or family physician**. Not all GPs, nor all endocrinologists, have experience in managing gender-affirming HT, and the expertise of the clinician should be the guiding factor in determining who prescribes and monitors your HT.

You may benefit from seeing other **allied health professionals**, such as **speech therapists**, or from medical specialists such as **plastic surgeons**. Not every TGD client will necessarily want to pursue these options, and you should discuss your individual goals with your prescribing doctor.

### Home affairs and gender marker

If you wish to update your gender marker on your birth certificate and ID document, the Department of Home Affairs requires **two letters from healthcare professionals,** which state that you have undergone **medical or surgical** gender reassignment. Either of these is sufficient; you do **not need to have had surgery** to update your gender marker.

Unfortunately, at present, gender markers in South Africa are binary – thus, an ID document can reflect either female or male gender; there is no unspecified or non-binary marker.

Your prescribing doctor can write one of your letters for the Department of Home Affairs and should be able to refer you to another healthcare provider to write the second letter.

If you wish to change your name, this process should be undertaken separate to updating your gender marker (either before or after). This **does not require supporting letters** from healthcare providers.

**Potential challenges with legal documents:** Some TGD persons have trouble with banking, registration as a student and writing examinations, registration of motor vehicles, and so on whilst they are waiting for their new documents. Some are accused of potential fraud because they do not look the same as the photograph in their identity document or their name may be different from the one on their qualification certificates. Changing one’s legal gender marker and names will take a while. You may want to consider how you will deal with it in the interim. You also may want to consider whether you need to change other documentation, for example your matric certificate.

Other important aspects to explore:

**Potential impact on emotions:** The impact of testosterone can be very diverse and individualised. Your mood may fluctuate. For example, often a TGD man may struggle to cry, and their emotions may become less intense. Some also experience increased irritability.

**Potential impact on relationships with family and significant others:** Have you thought about the possible impact on your relationship with significant others? If you are in an intimate relationship, this may change when you start on hormones, and relationship roles may need to be re-negotiated. A partner may grieve the loss of aspects of who you were and the way the relationship used to be. What are the possible impacts it can have on your family and how will you be able to deal with your family’s response? Have you considered the impact of the change of gender role in your family? Have you considered the impact of potential loss of fertility? Are there children that may be impacted and are the children prepared?

**Potential change in sexual orientation:** It is possible that your sexual orientation may remain constant or shift, either temporarily or permanently (e.g. shift in attraction or choice of sexual partners, widened spectrum of attraction and shift in sexual orientation identity).

**Potential impact on safety:** In some settings, the physical changes in hormones may have an impact on your safety, with people who do not fit into society’s expectations of male or female being at increased risk of violence.

**Potential impact on employment:** Some TGD persons experience discrimination in the workplace or struggle to obtain employment. This may be more difficult when your legal documents (e.g. identity document) and your appearance do not match.

**Potential grief and loss:** Some TGD persons experience a sense of loss. A TGD man may lose certain gender roles in the family.

**Taste changes:** Some TGD persons experience a change in their taste sensations, and their likes and dislikes of certain foods.

**Body odour change:** Some TGD persons experience a change in their body odour on hormone treatment.

**Appetite and sleeping patterns:** Often the TGD person will experience an increase of appetite. This could lead to weight gain. Sleeping patterns may also be affected.

### Masculinising hormone therapy

Masculinising HT is prescribed for assigned-female-at-birth clients who wish to masculinise. The backbone of masculinising HT is **testosterone** therapy. No additional medications are necessary to suppress oestrogen, as testosterone is able to do this alone.

The biggest concern with testosterone therapy is the **risk of liver and cardiovascular disease**. Testosterone use can adversely affect the liver, which is an organ vital to detoxifying the blood, and metabolising medications and dietary nutrients. Changes in testosterone levels have also been found to increase low-density lipoprotein (commonly known as ‘bad’) cholesterol and decrease high-density lipoprotein (commonly known as ‘good’) cholesterol. These changes in the metabolic profile can increase a client’s risk of heart attacks or strokes to levels similar to those seen in cisgender men.

**Table:** Comparison of testosterone preparations.

Type	Examples	Advantages	Disadvantages
Short-acting injection	Depo-testosterone	Accessible at most pharmacies Affordable Once-a-week dosing	Requires knowledge of injection technique Requires disposables (syringes, needles and alcohol swabs)
Long-acting injection	Nebido	Accessible at most pharmacies A single dose lasts approximately 3 months	Requires knowledge of injection technique Requires disposables Achieving the correct dose can be difficult with long dosing intervals
Topical gels or creams		No injection needed	Available only from compounding pharmacy Skin absorption varies between clients

Your doctor will discuss with you the various options for HT that are available and help you to decide which form of treatment is best for you.

#### Additional medications

For clients who wish to achieve **suppression of menstruation**, but have not performed so on testosterone alone, **progesterone may be added**.

Some clients will use **topical minoxidil** in order to achieve fuller facial hair growth.

#### Costs of hormone therapy

It is important to remember that not only do different clients have different needs in accessing HT but also that prices for medication may vary between different pharmacies, and that these prices may fluctuate over time. The majority of clients can expect to spend between R200 and R600 per month on HT. This does not include monitoring blood tests or doctor’s visits.

#### Changes that occur when using hormone therapy

The changes you will experience on HT often take some time to fully develop. Some of these changes are **reversible** and will **disappear should you discontinue HT**. Others are **irreversible** and will **persist even if you stop taking your hormones**.

The timeline for these changes to begin is variable; however, most of them will only reach their maximum degree after 3–5 years on HT.

#### Reversible changes

- gain of muscle mass and increased strength
- changes in body fat distribution, possibly associated with weight gain (increased fat deposition in the abdomen, and decreased fat in breasts, buttocks and thighs)
- coarser and thicker skin
- increased acne
- coarser and thicker body hair
- increased red blood cell count
- increase in sex drive
- changes in mood and emotional response (often initially an increase in irritability, amongst other emotions)
- cessation of menses and ovulation, and dryness of the genital tissues.

#### Irreversible changes

- hair loss or male pattern baldness may occur
- facial hair growth
- deepening of the voice
- enlargement of the clitoris
- infertility.

#### Limitations of hormone therapy

It is important to understand that there are certain features that HT cannot alter, which include the following:

- presence of breast tissue – HT can reduce fat deposition in the breasts and make them smaller; however, it will not result in a loss of actual breast tissue
- bone structure – HT will not change the structure of your pelvis or make you grow taller

#### Important risks associated with hormone therapy

As with any medication, HT carries with it certain risks. Some of these risks can be mitigated or reduced by lifestyle factors, whilst others are independent risks that cannot be altered.

It is important to be fully aware of the risks associated with HT before making the decision to start your treatment:

- high cholesterol or blood fats
- increased red blood cell count
- high blood pressure.

All of the above can lead to or worsen **cardiovascular disease,** or lead to **strokes.** These conditions can be life threatening.

#### Liver disease

**Psychiatric symptoms** include mood disturbances, anxiety or psychosis, especially if there are pre-existing mental health conditions. If you have been diagnosed with a mental health condition and/or use psychiatric medication, you need to discuss the starting of HT with your doctor. The use of hormones can interact with various medications and may have an impact on your mental health conditions.

Some of the risks mentioned are modified by other factors. Notably, cardiovascular and clot risks are worse in clients who

- smoke
- use alcohol
- have pre-existing medical conditions, such as diabetes, high blood pressure and high cholesterol.

### Fertility

Although not all clients become infertile on HT, and some might regain fertility if they stop HT, many may become irreversibly infertile. Hormone therapy is not a replacement for effective and responsible contraceptive use.

All clients considering starting on HT should consider using a Cryobank to preserve their genetic material, in case they wish to conceive genetically related children at a later stage. Even if this is not a priority for you at this stage in your life, please consider the possibility that your perspectives might change with time, and that it is ideal to store genetic material before starting with HT rather than trying to regain fertility once you are already on HT.

Your doctor can refer you to facilities that can aid in cryopreservation.

### Monitoring and follow-up

Your doctor will advise and guide you in monitoring your safety whilst you are on HT. Usually, this will involve regular check-ups and physical examinations, as well as certain blood tests.

At the outset, it is not uncommon for these evaluations to be performed monthly, whilst you are still achieving the correct hormonal balance for you. Later, once you are stable on treatment, these intervals might be extended to 6-monthly, or perhaps even annually. This schedule is different for every client.

If you decide to stop your HT, you should discuss this decision with your doctor. It can be dangerous to abruptly withdraw HT without adequate medical supervision.

### More information

Please remember that you can discuss any questions or concerns with your doctor at any time.

Information on self-injection technique can be found at: https://fenwayhealth.org/wp-content/uploads/2015/07/COM-1880-TGD-health_injection-guide_small_v2.pdf

### Informed consent for masculinising hormone treatment

I confirm that I have read and understand the information above.

I confirm that my doctor has told me about the effects of masculinising hormone treatment, including the more common or serious risks and side effects as mentioned above.

I understand that some of these effects may be permanent.

I understand that as part of my treatment plan, I shall take my medication as prescribed and have check-ups, including blood tests, as required.

My doctor has offered me adequate opportunity to ask any questions that I have regarding masculinising hormone therapy.

I hereby agree that my doctor starts/continues treating me with masculinising hormone therapy.
